# Families’ Perceptions of the Motor Development and Quality of Life of Their Children Aged 0–3 Years during Home Confinement Due to the COVID-19 Pandemic. A Descriptive Study

**DOI:** 10.3390/children8121149

**Published:** 2021-12-07

**Authors:** Alicia Oliva-Arnanz, Helena Romay-Barrero, Rita-Pilar Romero-Galisteo, Elena Pinero-Pinto, Cristina Lirio-Romero, Rocío Palomo-Carrión

**Affiliations:** 1 Department of Physiotherapy in Hospital Gregorio Marañón, 28007 Madrid, Spain; alicia.oliva.fisioterapia@gmail.com; 2 Department of Nursing, Physiotherapy and Occupational Therapy, Faculty of Physiotherapy and Nursing, University of Castilla-La Mancha, 45071 Toledo, Spain; Cristina.Lirio@uclm.es (C.L.-R.); Rocio.Palomo@uclm.es (R.P.-C.); 3 Pediatric-Unit, Hemi-Child-Research (GIFTO), UCLM, 45071 Toledo, Spain; 4 Department of Physiotherapy, Faculty of Science Health, University of Málaga, 29016 Málaga, Spain; rpromero@uma.es; 5 Department of Physical Therapy, Faculty of Nursery, Physiotherapy and Podiatry, University of Seville, 41004 Seville, Spain; epinero@us.es

**Keywords:** quality of life, home confinement, COVID-19, motor development, typical development, early age, pandemic

## Abstract

The child’s interaction with the natural environment allows different learning opportunities and favors their motor development, which may be affected after a period of environmental deprivation, a consequence of home confinement due to the COVID-19 pandemic. The main objective of the study was to analyze the different areas of motor development, as well as the quality of life of children aged 0 to 3 years old after home confinement by COVID-19 and the possible correlation between both variables, and the influence of parental stimulation on motor development during this time of exclusive interaction with the immediate environment (home and family). A descriptive study was performed. A simple and anonymous questionnaire was created for parents of children between 0 and 3 years old who lived in Spain during the period of home confinement due to COVID-19 (March to June 2020). The measurement instrument used was a questionnaire made in “Google Forms”, where the variables were collected: Motor development (measured through the Ages & Stages Questionnaire, ASQ3), Quality of life (assessed with the Pediatric Quality of Life Inventory, PedsQL) and other variables, such as stimulation, performed during home confinement. Eighty-eight questionnaires were validated. The highest score in the motor development domains were obtained in children 2–3 years old. The motor domain of children aged 2–3 years old that obtained the highest score was communication (M = 54.69 ± 10.03) and the highest score in the quality of life was obtained in children aged 0–1 years old (M = 85.47 ± 12.39), also acquiring the lowest score in the emotional domain in all age groups (0–1, 1–2 and 2–3 years old). The assessment of motor development and quality of life after home confinement due to the COVID-19 pandemic did not determine low values, so it would not have been affected during this period of lack of interaction with the natural environment. Emphasizing that the emotional aspect within quality of life was the lowest score, this indicates that children from 0 to 3 years old need more emotional support in situations of variability of daily routines and of family stress.

## 1. Introduction

Motor development can be defined as the “continuous change in motor aspects that takes place throughout the human life cycle” [1]. “Motor development is the progressive acquisition of functional abilities of the child, that reflect the maturation of the structures of the Central Nervous System that support them.” [2].

According to the Dynamic Systems Theory [1], the development of a new motor skill emerges from the relationship that the subject establishes with the activity that is being developed and with the context in which it takes place [1], and the importance of social relationships and the environment in promoting movement and motor development of the child is reflected [3,4,5,6]. Child’s development is a dynamic process and encompasses various domains, such as gross motor skills, fine motor skills, cognitive, language, problems-solving, and socio-emotional aspects [5,7], which are both interrelated and complex in themselves [5].

One of the situations that has led to social deprivation, as well as to a decrease in interaction of the child with the environment, and therefore to the reduction of their possibilities of learning and acquisition of motor milestones at an appropriate age, may have been the confinement situation due to the COVID-19 pandemic. In December 2019, after numerous cases of severe pneumonia in China (starting in Wuhan), the presence of the new Coronavirus was detected, which was called Severe Acute Respiratory Syndrome (SARS-CoV-2), and it was declared a pandemic in March 2020 by the World Health Organization [8,9]. A public health emergency caused by COVID-19 was considered, and a state of alarm was declared at the national level, on 14 March, in which immediate extraordinary measures were established such as the limitation of the freedom of movement of people, which included home confinement of the population, face-to-face educational activity suspended in all centers and levels of education, in addition to restrictions in commercial, cultural, hotel, restaurant and leisure activities, [10] and children could not go outside, though later they were allowed to accompany their parents during basic errands [11].

The state of alarm lasted until 21 June 2020, giving place to the “new normal” [12]. Confinement by COVID-19 has meant a radical change in our daily routines, and of course also in those of the child and adolescent population, causing deterioration in their emotional health [9]. Most parents and professionals consider that confinement had negative effects and changes in behavior [4,8,9,13]. Therefore, during the period of confinement, families had to adapt their work and family situation. Families faced countless stresses during confinement due to numerous job losses and difficulties in maintaining basic needs, such as food security and reliable childcare. All these stressors can increase psychological pressure on families [14]. Some of the emotional consequences of confinement in the general population have been boredom, social isolation, sleep disturbances, eating disorders and stress, also in children [15,16,17,18,19].

Due to all these stressors that were experienced during the home confinement situation, the condition of children at an early age may have been affected by the lack of interaction with the environment and by family stress. Thus, the main objective of this study was to analyze the different areas of motor development, as well as the quality of life, of children aged 0 to 3 years old after home confinement due to COVID-19 and the possible correlation between both variables, as well as the influence of parental stimulation on motor development during that time of exclusive interaction with the immediate environment (home and family).

## 2. Materials and Methods

### 2.1. Study Design

This was a descriptive study.

### 2.2. Inclusion/Exclusion Criteria

To be included in the study, the participants must have filled out a questionnaire and it was required to reside in Spain during the home confinement (From 14 March 2020 to 21 June 2020) during theCOVID-19 pandemic, to have children with typical development who had attended kindergarten before home confinement or who had not, children with corrected ages between 0–3 years, and children born at term and late preterm (34–37 weeks of gestation) who did not have any developmental delay, or specific medical follow-up, or rehabilitation.

The study excluded children with a diagnosis of any pathology or syndrome that leads to delayed motor development, families that have not experienced a full period of confinement, families of children who were born during or after confinement (born from March 2020), and families of children who were already three years old at the beginning of the confinement.

### 2.3. Participants

The study was composed of 88 families who lived in Spain during home confinement (from 14 March 2020 to 21 June 2020) due to the COVID-19 pandemic, whose children were aged 0–3 years old. The sample recruitment was carried out through nursery schools and pediatric health centers from different Autonomous Communities of Spain, whom were contacted to distribute the questionnaire and disseminate it to the families. In addition, social networks were used to expand its dissemination.

### 2.4. Outcome Measures

The main study variables analyzed in the research were the motor development and quality of life of the children who met the established inclusion criteria.

#### 2.4.1. Motor Development Was Measured through the Ages & Stages Questionnaire or the Ages and Stages Questionnaire (ASQ3)

This consists of a Global Screening tool, designed by Squires and Bricker at the University of Oregon [20]. It is used internationally to evaluate the psychomotor development of children, being a questionnaire aimed at parents, in which the motor development of the child is evaluated in five different domains: Communication, Gross Motor, Fine Motor, Problem-solving and Socio-individual, for children between 2–66 months [21,22,23].

Each domain has six questions that can be answered by Yes, Maybe, Not Yet, which are transformed into numerical scores of Yes = 10, Maybe = 5 and Not Yet = 0. Therefore, in each domain a maximum score of 60 points can be obtained. The limit scores that determine the existence of a delay in motor development for each domain are specified by age in the ASQ3 Scale. In the case of the ages studied in the our research, the ASQ3 scale shows that these limit scores would be: 0–1 years old: Communication: 15.64; Gross Motor: 21.49; Fine Motor: 34.50 Problems-solving: 27.32 and Socio-individual: 21.73, 1–2 years old: Communication: 25.17; Gross Motor: 38.07; Fine Motor: 35.16; Problems-solving: 29.78 and Socio-individual: 31.54 and age from 2–3 years old: Communication: 30.99; Gross Motor: 36.99; Fine Motor: 18.07; Problems-solving: 30.29 and Socio-individual: 35.33.

This has been cross-culturally validated in several countries, Spain being one of them (in Galicia) [24,25]. The scale has a reliability of 92%, a sensitivity of 87.4%, and a specificity of 95.7% [25]. Its administration is intended to detect if there is a delay in development in the population that has experienced confinement, or to verify if any dimension that reflects this scale has been affected to a greater extent.

#### 2.4.2. Pediatric Quality of Life Was Measured through the Pediatric Quality of Life Inventory or Pediatric Quality of Life Questionnaire (PedsQL)

This consists of a questionnaire for children between 2–18 years old, specifically the version that has been used for the sample between 2–3 years old, which evaluates three dimensions: Physical health and activities (5 items), Emotional state (4 items) and Social activities (3 items) [26]. It has great validity in measuring pediatric quality of life [26,27].

A series of predetermined items are collected within each dimension, with the parents evaluating the frequency of problems that their children might present on a scale 0–4 for each item: (0: Never, 1: Almost never, 2: Sometimes, 3: Frequently and 4: Almost always).

For the youngest (0–1 year and 1–2 years) a version of this Scale adapted for babies has been used that evaluates the items in the same way as explained. On the scale for children aged 0–24 months, five dimensions are evaluated: Physical functioning (6 items), Physical symptoms (10 items), Emotional functioning (12 items), Social functioning (4 items) and Cognitive functioning (4 items). (29). For children aged 13–24 months, the same dimensions are maintained, but there is a change in the number of items: Physical functioning (9 items), Physical symptoms (10 items), Emotional functioning (12 items), Social functioning (5 items) and Cognitive functioning (9 items) [28].

For each item, parents answer on a 0–4 scale: (0: never a problem; 1: almost never a problem; 2: sometimes, 3: frequently, 4: almost always).

The minimum score in each domain and for global quality of life can be 0 points and the maximum score can be 100 points for both 0–2 years old and 2–3 years old, as a numeric score in the scale 0–4: 0 = 100 points; 1 = 75 points; 2 = 50 points; 3 = 25 points and 4 = 0 points.

### 2.5. Data Collection

The data collection of the variables “motor development and quality of life” was carried out shortly after the home confinement with a simple online questionnaire, through “Google Forms”. To complete the questionnaire, the informed consent of the family was required, with their agreement to participate in the research. All data collected was anonymous, maintaining the individual integrity of the person and data protection at all times. Once the family had completed the questionnaire, the data was uploaded electronically. Only the study investigators had access to identifiable data.

The questionnaire consists of, firstly, a consent to be part of the study in which the participants had to check a box to be able to take the questionnaire in which they confirmed that they had read and understood the procedure described and agreed to participate voluntarily. Then, global and sociodemographic characteristics of the study participants, families and children were collected:

- Initial data of the child: Sex, age, Autonomous Community, prematurity, diagnosis of any pathology.
- Data before and during confinement: previous attendance at nursery school, and performance of stimulation work during confinement
- Questions that collect the opinions of the parents: if they believe that the confinement had affected the motor development of their child, the importance of the environment in the development of the child and if they felt concerns about whether the confinement affected the motor development of their child (son/daughter), with selectable response options: Very little-Little-Fair-Fairly-Much.

This first part was filled in by all participants equally. Subsequently, depending on the age range of the child when the questionnaire was completed, they were directed to its corresponding section in the scales mentioned above (ASQ3 and PedsQL).

### 2.6. Ethical Considerations

#### 2.6.1. Ethical Approval

The study complies with the Helsinki regulations, as well as with the Spanish Law on Data Protection and Guarantee of Digital Rights, of December 2018. The study has also been approved by the Ethics and Experimentation Committee of the CEU-San University Pablo (Reference no. 480/21/TFM).

#### 2.6.2. Informed Consent

Informed consent was obtained via initial contact with the families by electronically, sending the questionnaire. They received information on the objectives established regarding the completion of the questionnaire. If they did not receive primary education, the fact sheet could be read by someone else and would be completed based on what the family said. Then, they signed an informed consent form that would approve the use of their data for research purposes and dissemination of results.

#### 2.6.3. Confidentiality

The anonymity of the participants was preserved and complied with by the precepts of the Law on Protection of Personal Data in force in Spain. The principal investigator for this project was the only person with access to the dataset.

### 2.7. Data Analysis

The data were coded, tabulated and statistically analyzed using the Statistical Package SPSS version 25. A descriptive analysis of the variables was made. An ANOVA test was used to check if there were statistically significant differences between the quality of life and the different age groups, between the gross and fine motor development domains and the different age groups and finally between the gross motor, fine motor and communication domains, with the possibility of receiving, or not, stimulation by parents or professionals during the confinement period. If statistically significant differences were obtained, a multiple comparisons test would be performed. Finally, Pearson’s linear correlation coefficient was calculated to analyze the correlation between the quality of life variable and the different domains of motor development. The results are expressed as mean and standard deviation (SD), considering their level of statistical significance, with an intraclass correlation coefficient of 95% and a *p* value equal to or less than 0.05.

## 3. Results

**Figure 1:**
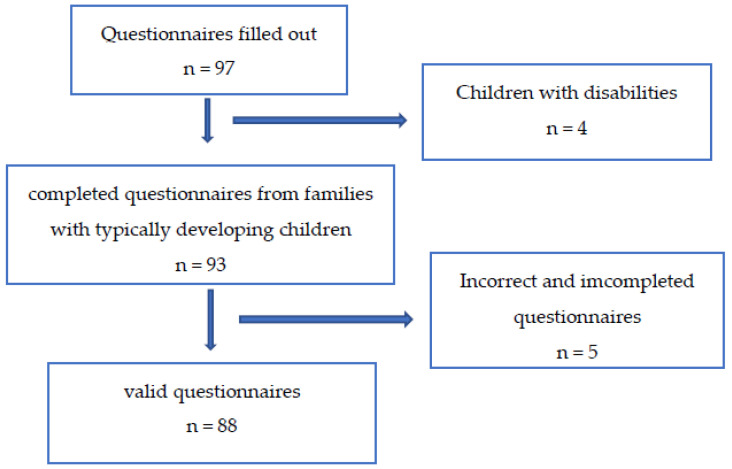
Flow chart of questionnaire collection.

The questionnaire was completed correctly by 88 families who had completed primary studies and had a stable economic situation (Figure 1). Families lived at home during confinement in Spain, with children with ages between 0–3 years: age range of 0–1 year: 34 children (38.6%), age range of 1–2 years: 41 children (46.6%), and age range of 2–3 years: 13 children (14.3%). The sample was formed by 51.1% males (age range-0–1 year: *n* = 16, age mean = 8.5 months ± 1.43; age range-1–2 years: *n* = 21, age mean: 20 months ± 1.89 and age range-2–3 years: *n* = 8, age mean: 30 months ± 1.45) and 48.9% females (age range-0–1 year: *n* = 18, age mean = 10 months ± 1.29; age’s range-1–2 years: *n* = 20, age mean: 18 months ± 1.7 and age range-2–3 years: *n* = 5, age mean: 32 months ± 1.56), from different communities in Spain (Community of Madrid (52%), Valencian Community (1%), Castilla La Mancha (7%), Castilla y León (7%), Aragon (1%), Navarra (9%), Galicia (18%) and Andalucía (5%). Only three children had been premature.

The results regarding the different domains of motor development represented the highest scores for children with an age between 2–3 years (Table 1). The motor development domain of the age range of 2–3 years old that obtained the highest score was communication with a mean of 54.69 ± 10.03, with respect to the domain with the lowest score, which was the fine motor domain (M = 46.54 ± 10.87). However, in children with an age of 1–2 years, the highest score was obtained in the gross motor domain, the lowest being perceived in the socio-individual area, as also occurs for ages 0–1 years (Table 1).

The highest score for quality of life was obtained in the age of 0–1 years (M = 85.47 ± 12.39) and was very similar in male and female (Table 2). The Physical Health dimension for quality of life is the area with the highest score in the age range 1–2 years old with a mean of 90.56 ± 10.46 and 2–3 years old with a mean of 86.92 ±14.65 (Table 3 and Table 4). The quality of life domain with lowest score was the emotional area in all age’s ranges: 0–1 years old: M = 71.35 ± 20.88, 1–2 years old: M = 67.18 ± 20.65 and 2–3 years old: M = 63.94 ± 13.78 (Table 3 and Table 4).

In the answers to the questionnaires, more than 40% of the parents gave importance to the environment in the acquisition of the motor development of their children, but less than 5% of the families thought that home confinement could influence motor development of their children. A percentage of 62.5 attended kindergarten before the period of confinement, while 37.5% did not attend. Parents generally performed stimulation work with their children during confinement: 67% with ideas from parents and 8% guided by a kindergarten’s professional, while 25% did not perform any type of stimulation. No statistically significant differences were obtained in the Communication domain with any stimulation type (guided by a kindergarten professional or by the parents’ ideas) or no stimulation (*p* = 0.42), Gross Motor domain with any stimulation type (guided by a kindergarten professional or by parents’ ideas) or no stimulation (*p* = 0.68) and Fine Motor domain with any stimulation type (guided by a kindergarten professional or by parents’ ideas) or no stimulation (*p* = 0.73).

There was no association between the three age groups with the variables: Quality of life (*p* = 0.06), Gross Motor domain into ASQ3 (*p* = 0.357), Fine Motor domain into ASQ3 (*p* = 0.521), Problem-solving domain into ASQ3 (*p* = 0.451) and Communication domain into ASQ3 (*p* = 0.419). There was no correlation between quality of life and the different domains of the ASQ3-motor development scale (*r* < 0.2 and *p* > 0.05). However, there was a correlation between the different domains of motor development (ASQ3 scale): Gross Motor domain and Communication domain (*r* = 0.304, *p* = 0.004); Communication domain with Fine Motor domain (*r* = 0.372, *p* <0.001), Communication domain with Problems-Solving domain (*r* = 0.535, *p* < 0.001), Communication domain with Socio-individual domain (*r* = 0.524, *p* < 0.001), Gross Motor domain with Fine Motor domain (*r* = 0.453, *p* < 0.001), Gross Motor domain with Problems-Solving domain (*r* = 0.465, *p* < 0.001), Gross Motor domain with Socio-individual domain (*r* = 0.387, *p* < 0.001), Fine Motor domain with Problem-Solving domain (*r* = 0.391, *p* < 0.001), Fine Motor domain with Socio-individual domain (*r* = 0.347, *p* < 0.001), and Problem-Solving domain with Socio-individual domain (*r* = 0.452, *p* < 0.001).

## 4. Discussion

The environment offers multiple possibilities for interaction with it, which influences the child’s motor development. In fact, the acquisition of new motor skills is the result of the activity that takes place and the context in which it is carried out, so the children’s environment is important for their development and generation of new skills [1]. In the present study, 50% of families consider the influence of the environment on motor development as a very important factor, 36.4% consider it quite important, 12.5% regular, and only 1% of those surveyed consider that it has very little or little importance. These results coincide with those of the study of Adolph and Hoch [5], which concludes that motor development is closely related to the environment, since the environment can provide opportunities for the development of new capacities or abilities in children.

This interaction with the natural environment can be verified in our study, in which children with an age range of 2–3 years obtained better scores in motor development than children in the age ranges of 0–1 and 1–2 years. This may suggest that children at 2–3-years old had greater opportunities to interact with the environment before home confinement, to acquire new skills and to promote their motor development at a more complex level, acquiring more communication structures.

Of parents in the present study, 86.36% consider that they felt a lot of concern about the development of their children at the time of confinement, but, nevertheless, 65.9% consider that the home confinement influenced the child only little or very little. Alarming scores were not obtained within motor delay in the population of children from 0 to 3 years old, which could be due to the fact that, despite contact with the external environment being restricted, the children had the opportunity to interact within their most immediate environment, such as home and family. Perhaps if it had been a population with a pathology that implied a developmental delay, there would have been differences in the response of children with typical and atypical development within the domains of motor development. Therefore, the concern for the development of children could be caused by other factors, other than the confinement itself, a consequence of a possible general concern of the parents regardless of the surrounding circumstances [14,16]. There are previous studies on how families have experienced confinement, mainly at a psychological level, presenting concerns, nightmares, sleep and appetite alterations, lack of attention, attachment, etc., [3,14,16,17,18] but no studies have been found on concern for children’s motor development after a modified environment during a period of forced stay at home and of a stressful nature, and in which the children missed opportunities to innovate in spaces other than the home, and to create social relationships outside the family nucleus.

Regarding the type of stimulation received, results obtained showed that parents who did not perform any type of stimulation with their children, or used their own resources, had lower scores in Gross Motor Development than those guided by a professional; nevertheless, in the Communication domain, even those who did not perform any type of stimulation obtained better scores. However, in the fine motor area, those who performed exercises suggested by parents’ ideas, or even did not, had better scores than those guided by a professional. Therefore, the role of the professional in the gross motor development of children should be highlighted; and it can be justified that novel fine motor activities have been prevalent among those proposed by parents during confinement, easier to do at home due to space and conditions, one of the great limitations during the pandemic [18,29]; coincides with the findings in the study by Moore S.A. et al. [29], where the activities most carried out at home during confinement were arts and crafts (12.9%), puzzles and games (11.3%) and video games (10.2%).

In the comparison of results between Sarmiento et al. [25] and our study, slightly lower scores were observed regardless of age in the fine motor and socio-individual domains, and similar scores in problem-solving, gross motor and communication domains, except at the age of 12 months, where it is notable that lower scores are obtained in areas such as communication and problems-solving. Considering that the study was carried out one year after the onset of the pandemic, children evaluated with an age of 12 months would correspond to newborn children at the time of confinement, and at the time of the birth of a child a family stress situation also occurs [6] which, added to the stress of the pandemic, could have had greater repercussions on children, coinciding with the result of the study by Orgilés et al. [9], which concluded that as the pandemic became more complicated, family stress increased, having a greater impact and repercussion on children.

According to the research performed, 50% of parents thought that physical and emotional health domains and quality of life would be affected in their children by confinement [18]. This corresponds with the findings obtained in previous studies on confined childhood in which it is concluded that confinement could affect the health and well-being of children and parents and the living conditions of families (family and social well-being), so that home confinement could have an impact on the quality of life of small children [18,30]. According to the results obtained in the present study, there are no data that represent that this had affected to the quality of life in children that are 0–3 years old, having a high score, but the data supported that the quality of life is lower in older children (2–3 years) [3,14,16,17,18]. This could have been a consequence of the lack of social interaction and emotional support, domains that obtained the lowest scores for quality of life of children aged 2–3 years, which suggests that at this age there is more repercussion on emotional well-being of the presence of the external environment, playing with other children and interaction with adults other than the parents, which would influence their emotional state and therefore their overall quality of life [14].

Children have had to struggle with substantial adjustments to their routines, like kindergarten and childcare closures, home confinement, and social distancing, and preventive measures could impact their sense of structure, predictability, and security [30]. Children, including infants and toddlers, observe their environments and people and, in this regard, they react to the stress of their parents and other caregivers, peers, and community members [14,15,30]. Being caught in the COVID-19 outbreak pandemic is considerably stressful for children, can lead to traumatic stress, and endangers the children’s sense of security, leave them helpless and susceptible [30]. Thus, the lowest score in the quality of life for all children was observed in the “Emotional functioning” domain, which could be correlated with the feelings previously described by children during confinement, [3,14,16,17,18], becoming more aware of the situation the older they are. This is backed up by the result of the study by Erades and Morales [19] in Spain, in which emotional reactions were the most frequent (69.6%) during confinement. At the social level, in the age range 0–2 years old, high values were obtained, but in the age range of 2–3 years the score obtained is the lowest. This can be related to the usual environment of the children, since during the confinement there was social isolation (among other effects), but the social relationships of a child in the age range 0–2 years old are not as evident as in older ones, and therefore children between the ages of 2–3 years old could have been affected by their social activity during confinement [15]. This differentiation in the quality of life within the age ranges could be influenced by the child’s need to experiment with the environment in order to acquire global functioning and optimization of the quality of life. The opportunities for interaction and exploration of the natural environment are greater from 18 months of age when the child has a more precise acquisition of gait and manual ability that allows him/her to be able to acquire functional strategies that will enhance his/her motor development and quality of life. Correlating this with motor milestones, those that favor ambulation are established at 18–24 months, [2,3,7] and the acquisition of these skills to transport in space can benefit children to expand the environment they want to discover. This is appreciated in our study in the correlation between all domains and motor development. Therefore, children at age 2–3 years old who also begin to interact more with their peers, would reduce their social relationships and interaction with their environment, which could limit their learning capacities, and therefore their quality of life. However, the environment of children with an age range of 0–1 years old was modified by confinement, and perhaps their day-to-day life in a situation without confinement would have been similar in terms of interaction with the natural environment.

Quality of life can be correlated with different social and personal aspects that were studied in other investigations [18,31], and not with motor development, since there was no correlation between the different domains of motor development and the child’s quality of life studied in our research.

As limitations of the study, it could be noted that it was performed post-confinement and with a small sample, since in other studies a larger sample was used and therefore the data obtained could be more representative of the general population in order to compare data from children with typical and atypical development. The results should be interpreted with caution, since the age ranges do not cover a large proportion of the population, so it is not possible to generalize.

A strength of the present study is the focus on different aspects such as the assessment of motor development, the quality of life after home confinement and how it was experienced during that period of time, as well as the parents’ interaction with stimulation of their children from 0 to 3 years old, not previously studied.

## 5. Conclusions

The assessment of motor development and quality of life in children (0–3 years old) after home confinement due to the COVID-19 pandemic did not determine low values, so it would not be affected during this period of lack of interaction with the natural environment. It is emphasized that the emotional aspect within quality of life showed the lowest, which, which would indicate that children from 0 to 3 years old need more emotional support in situations of variability of routine and family stress during home confinement.

## Figures and Tables

**Table 1:** Scores for each age in the different motor development domains in the ASQ3 scale.

Motor Development Domains	Age from 0 to 1 Years Old (*n* = 34)	Age from 1 to 2 Years Old (*n* = 41)	Age from 2 to 3 Years Old (*n* = 13)
Communication	41.20 (12.32)	45.60 (12.67)	54.69 (10.03)
Gross motor	49.12 (10.76)	51.46 (8.3)	53.08 (8.3)
Fine motor	43.68 (11.95)	43.86 (12.98)	46.54 (10.87)
Problem-solving	35 (13.34)	41 (13.20)	52.71 (9.31)
Socio-individual	28.25 (11.65)	39.01 (10.27)	51.63 (9.52)

Scores in the motor development domains from minimum from 0 points to maximum to 60 points. Values expressed in mean and Standard Deviation (SD).

**Table 2:** Results from the PedsQL scale by age groups and sex.

Age groups	0–1 years (*n* = 34)	85.7 (12.39)
	1–2 years (*n* =4 1)	83.93 (10.37)
	2–3 years (*n* =1 3)	76.48 (13.70)
	0–3 years (*n* = 88)	83.42 (11.94)
Sex	Male (*n* = 45)	83.76 (10.79)
	Female (*n* = 43)	83.07 (13.16)

Scores are shown in a scale in PedsQL from 0 to 100. Values are expressed in mean and Standard Deviation (SD).

**Table 3:** Results from PedsQL dimensions in the age ranges of 0–1 years old and 1–2 years old.

Quality of Life Dimensions	0–1 Years Old (*n* = 34)	1–2 Years Old (*n* = 41)
Physical functioning	89.4 (12.57)	86.04 (13.07)
Physical Health	88.16 (1093)	90.56 (10.46)
Emotional Functioning	71.35 (20.88)	67.18 (20.65)
Social functioning	90.81 (19.54)	88.17 (15.60)
Cognitive functioning	87.68 (20.09)	88.48 (14.25)

Scores are shown in a PedsQL scale from 0 to 100. Values are expressed in mean and Standard Deviation (SD).

**Table 4:** Results from PedsQL dimensions in the age range 2–3 years old.

Quality of Life Dimensions	2–3 Years Old (*n* = 13)
Physical Health	86.2 (14.65)
Emotional	63.94 (13.78)
Social activity	77.57 (23.91)

Values expressed as means and standard deviation (SD).
